# Catechin Suppresses an Array of Signalling Molecules and Modulates Alcohol-Induced Endotoxin Mediated Liver Injury in a Rat Model

**DOI:** 10.1371/journal.pone.0020635

**Published:** 2011-06-03

**Authors:** Sushma Bharrhan, Ashwani Koul, Kanwaljit Chopra, Praveen Rishi

**Affiliations:** 1 Department of Microbiology, Basic Medical Sciences Block, Panjab University, Chandigarh, India; 2 Department of Biophysics, Basic Medical Sciences Block, Panjab University, Chandigarh, India; 3 Pharmacology Research Laboratory, University Institute of Pharmaceutical Sciences, UGC Centre of Advanced Study, Panjab University, Chandigarh, India; University of Hong Kong, Hong Kong

## Abstract

Induction of nuclear factor kappa B (NF-κB)-mediated gene expression has been implicated in the pathogenesis of alcoholic liver disease through enhanced production of reactive oxygen species and pro-inflammatory mediators. The present study was carried out to investigate the role of catechin as a chain breaking inhibitor against experimental alcoholic liver injury. Rats were administered 35% v/v ethanol orally at a dose of 10 g/Kg/day for two weeks, followed by 14 g/Kg/day for 10 weeks. Catechin (50 mg/Kg) was co-supplemented after 4 weeks of alcohol treatment till the end of the dosing period. Following chronic alcohol exposure, rats developed endotoxemia and severe pathological changes in the liver such as pronounced fatty change, vacuolar degeneration and inflammation. These changes were accompanied by activation of NF-κB and induction of inflammatory and cytotoxic mediators leading to increased level of tumor necrosis factor-alpha, enhanced formation of malondialdehyde in the liver followed by drastic alterations in the hepatic antioxidant defense systems. Additionally, nitrite levels and lactate dehydrogenase activities were also significantly elevated on chronic alcohol consumption. Alcohol exposure also increased the number of micronucleated cells indicating that alcohol abuse may again be associated with the nuclear changes. Supplementation with catechin ameliorated the alcohol-induced liver injury by downregulating the endotoxin-mediated activation of initial signalling molecule NF-κB and further going downstream the signalling cascade including tumor necrosis factor-alpha, nitric oxide and reactive oxygen species and by enhancing the antioxidant profile. These observations correlated well with the histological findings. Moreover, a remarkable decrease in the percentage of micronucleated cells was observed with catechin supplementation indicating an apparent protection against alcohol-induced toxicity. These findings suggest that catechin may alleviate experimental alcoholic liver disease by suppressing induction of NF-κB, a key component of signalling pathway, thus forming a pharmacological basis for designing novel therapeutic agents against alcohol induced endotoxin-mediated liver injury.

## Introduction

Alcohol abuse remains a global social evil associated with a large number of clinical problems such as alcoholic liver disease (ALD) [1]–[3]. Regular consumption of alcohol can cause various hepatic abnormalities ranging from steatohepatitis to cirrhosis and hepatocellular carcinoma [4], [5]. Since no therapy except orthotopic liver transplantation for end stage liver disease is available, abstinence from chronic consumption of alcohol is the only way to avoid this dreadful pathology [2], [3], [5].

In recent years, it has become increasingly evident that alcohol ingestion facilitates the absorption of gut-derived endotoxin from the small intestine resulting in an increased level of endotoxin in the systemic circulation [6]. The endotoxin, thus released induces a signalling cascade leading to the activation of transcription factor NF-κB. Following activation, NF-κB gets translocated to the nucleus and causes rapid gene induction resulting in the expression of inflammatory mediators, including cytokines (particularly TNF-α, IL-6, IL-12), chemokines, lipid mediators, inducible nitric oxide synthase (iNOS), enzymes such as cyclooxygenase-2 and adhesion molecules [7]–[9]. TNF-α further stimulates the production of reactive oxygen species (ROS) and reactive nitrogen intermediates (RNIs) by the activated cells causing liver damage due to oxidative stress [10], [11].

Numerous interventions, such as intake of antioxidants, have been put forward to counteract/combat the oxidative stress due to alcohol consumption [12]–[14]. Among them, flavonoids have drawn interest of many researchers [15]–[19]. These are phenolic phytochemicals that constitute substantial part/portion of the non-energetic part of the human diet and are thought to promote optimal health, partly via their antioxidant effects in protecting cellular components against ROS and RNIs [20]. Flavonoids have been reported to be chain-breaking inhibitors of the peroxidation process, scavenging intermediate peroxyl and alkoxyl radicals [21]–[23]. Amongst them, catechins are naturally occurring polyphenolic compounds, which are found in abundance in green tea [24], [25]. Tea polyphenols have been shown to possess numerous biological functions, including potent antioxidant and anti-inflammatory properties [26], [27]. These have also been reported to protect against alcohol-induced liver injury in rats [15], [17], [18]. Although, catechin did not significantly improve alcoholic liver diseases in limited human clinical trials done back in 1980s [28], [29], extensive evaluation of catechin effects at higher doses in long-term trials has not been carried out. Moreover, there is no information on the *in vivo* role of catechin as a chain breaking inhibitor against oxidative stress generated due to alcoholic liver injury. The present study thus delineates the mechanism of inhibition of the signalling cascade involved in this particular clinical manifestation.

## Materials and Methods

### Ethics Statement

The experimental protocols were approved by the Institutional Animal Ethics Committee (Approval ID: 1-12/IAEC dated 3.09.2009) of the Panjab University, Chandigarh, India (Registration number: 51/1999/CPCSEA) and performed in accordance with the guidelines of Committee for the Purpose of Control and Supervision of Experiments on Animals (CPCSEA), Government of India, on animal experimentation. All efforts were made to minimize the suffering of animals.

### Agents

Absolute ethanol (99.9%) was purchased from Brampton, Ontario. Catechin hydrate was purchased from Sigma Aldrich Chemicals, St. Louis, MO, USA. The preparations were made fresh every time before the commencement of the experiment. Catechin hydrate was dissolved in warm distilled water and administered by oral gavage. All other chemicals were of analytical grade.

### Animals

Female Wistar rats (200–250 g) were procured from Central Animal House, Panjab University, Chandigarh (India). The animals were housed under standard laboratory conditions, maintained on a 12∶12 h light:dark cycle and had free access to food (Ashirwad Industries Pvt Ltd, Punjab, India) and water *ad-libitum*.

### Experimental design

After an acclimatizing period, rats were randomly divided into following four groups each comprising of 10–12 rats. **(i)** **Control group:** Rats in this group were given distilled water orally; **(ii)** **Alcohol group (Alc):** Rats were administered 10 g/Kg body weight/day of 35% (v/v) ethanol by oral gavage in double distilled water for two weeks. Thereafter, the dose was increased to 14 g/Kg body weight/day and was continued for 10 weeks; **(iii)** **Catechin supplemented group (CT** ***per se*** **):** Catechin (CT) at a dose of 50 mg/Kg body weight was administered orally to rats for 8 weeks to see the *per se* effect; **(iv)** **Alcohol fed and catechin supplemented group (Alc + CT):** After 4 weeks of alcohol administration, rats were supplemented with catechin (50 mg/Kg body weight/day) by oral route 1 h before alcohol administration daily till the end of the dosing period (i.e. for rest of the 8 weeks). At the end of the experimental period (after 12 weeks), the rats were sacrificed by cervical dislocation. Livers were removed quickly, rinsed in cold phosphate buffer saline (0.05 M, pH 7.4) and stored at −62°C till further use.

### Measurement of blood alcohol

10 weeks after initiation of alcohol administration, blood was taken from the tail vein 1.5 h and 2.5 h after gavage. Blood alcohol levels (BAL) were measured using the alcohol dehydrogenase kit from Sigma Chemical Co., U.S.A.

### Plasma Endotoxin Assay

Endotoxin level in the plasma samples was measured using ToxinSensor Chromogenic LAL Endotoxin Assay Kit (GenScript USA Inc.). Briefly, 0.1 ml plasma was incubated with 0.1 ml Limulus amebocyte lysate (LAL) at 37°C for 45 min. After several subsequent reactions, the samples were read spectrophotometrically at 545 nm. The plasma endotoxin levels were calculated against a standard curve of endotoxin (*E. coli* 0113:H10) concentrations of 0.1, 0.04, 0.02, 0.01 and 0.005 EU/ml.

### Markers of liver damage

#### Assessment of liver function

Alanine aminotransferase (ALT) and aspartate aminotransferase (AST) enzyme activities in serum were determined using ERBA test kits (ERBA Diagnostics, Mannheim, Germany). Alkaline phosphatase (ALP) was estimated using Enzopak Diagnostic kit (Reckon Diagnostics, India).

#### Liver histology

Liver tissues removed aseptically from the animals were cut into small pieces and fixed in 10% buffered formalin. Samples were processed, stained with hematoxylin-eosin and examined under the light microscope. Histological interpretation was done by Dr. B. N. Datta, Ex-Professor of Pathology, Post Graduate Institute of Medical Education and Research, Chandigarh (India).

### Mechanistic studies

Livers removed aseptically from the rats were rinsed in 0.05 M phosphate buffer saline (pH 7.4) (PBS). A 25% (w/v) tissue homogenate was prepared in PBS using a Potter Elvehjen homogenizer. An aliquot of the liver homogenate was used for the estimation of lipid peroxidation and reduced glutathione levels. For the estimation of superoxide dismutase, catalase, glutathione reductase, glutathione peroxidase and lactate dehydrogenase activities, post mitochondrial preparation was made. For this, the liver homogenates were centrifuged at 11 269× *g* for 20 min at 4°C. The supernatants thus obtained were called as the post mitochondrial supernatants (PMS).

### Assessment of Oxidative Stress

#### Extent of peroxidative liver damage

The quantitative measurement of lipid peroxidation in liver was performed according to the method of Wills [30] as described earlier [31]. The results were expressed as nanomoles of malondialdehyde (MDA) per milligram of protein, using the molar extinction coefficient of chromophore (1.56×10^5^ M^−1^ cm^−1^). The protein content of tissue homogenates was calculated according to the method of Lowry et al. [32].

#### Estimation of hepatic reduced glutathione (GSH) levels

Reduced glutathione (GSH) levels in the liver homogenates were estimated according to the method of Jollow et al. [33]. The results were expressed as micromoles of GSH per milligram of protein, using the molar extinction coefficient of 5′-thiobis 2-nitrobenzoic acid (13600 M^−1^ cm^−1^).

#### Measurement of hepatic superoxide dismutase (SOD) activity

SOD activity was assayed according to the method of Kono [34] and was expressed as units of SOD per milligram of protein where one unit of activity was defined as the amount of SOD required to inhibit the rate of reduction of NBT by 50%.

#### Measurement of hepatic catalase activity

The catalase activity was assayed by the method of Luck [35] and was expressed as millimoles of H_2_O_2_ decomposed per min per mg of protein using the molar extinction coefficient of the chromophore (0.0394 mM^−1^ cm^−1^).

#### Measurement of hepatic glutathione peroxidase (GPx) activity

GPx activity was measured by the coupled assay method described by Paglia and Valentine [36]. Briefly, 1.0 ml of the reaction mixture contained 50 mM sodium phosphate buffer (pH 7.0) containing 1 mM EDTA, 0.24 U/ml yeast glutathione reductase, 0.3 mM reduced glutathione, 0.2 mM NADPH, 1.5 mM H_2_O_2_ and 25.0 µl of PMS. The reaction was initiated by adding NADPH and its oxidation was monitored at 340 nm by observing the decrease in OD/min for 3 min. One unit of enzyme was defined as nmol of NADPH consumed/min/mg protein based on extinction coefficient of 6.22 mM^−1^cm^−1^.

#### Measurement of hepatic glutathione reductase (GR) activity

GR was determined by the procedure described by Carlberg and Mannervick [37]. The reaction mixture (final volume 1.0 ml) contained 0.2 M sodium phosphate buffer (pH 7.0), 2 mM EDTA, 1 mM oxidized glutathione (GSSG) and 1.2 mM NADPH. The reaction was started by adding 20.0 µl of PMS and the enzyme activity was measured indirectly by monitoring the oxidation of NADPH following the decrease in OD/min for a minimum 3 min at 340 nm. One unit of enzyme activity was defined as nmol NADPH consumed/min/mg protein based on extinction coefficient of 6.22 mM^−1^cm^−1^.

### Measurement of lactate dehydrogenase (LDH) activity

LDH activity in serum and PMS samples was measured by recording the rate of oxidation of NADH by sodium pyruvate at 340 nm, according to the method of Bergmeyer et al. [38]. One unit of enzyme activity was defined as the amount of enzyme catalyzing the oxidation of 1 µmol of NADH per min based on extinction coefficient of 6.22 mM^−1^cm^−1^.

### Estimation of nitrite levels

Nitrite concentration was determined by Griess reaction, as described by Green et al. [39]. For this, 100 µl aliquots of serum or PMS were mixed with 100 µl of Griess reagent (0.1% naphthylethylene diamine dihydrochloric acid and 1% sulphanilamide in 5% phosphoric acid) and incubated at room temperature for 10 min. Absorbance was measured at 546 nm. Nitrite levels in all the samples were quantified according to the standard graph of sodium nitrite (in the range 1–10 µmoles/ml).

### Liver TNF-α assay

Assay for TNF-α was performed by ELISA in the liver homogenates using commercially available cytokine assay kit (R&D Systems, USA) according to the manufacturer's instructions. Briefly, standards and test samples were dispensed in the 96 well microtitre plates pre-coated with monoclonal antibody specific for rat TNF-α. To each of the designated wells, 50 µl of assay diluent was added, the plates were sealed with acetate plate sealer and incubated at room temperature for 2 h. Plates were then washed five times with the wash buffer and 100 µl of rat TNF-α conjugate was dispensed into each well. Plates were again sealed and incubated at room temperature for 2 h, after which they were washed five times with the wash buffer and 100 µl of substrate solution was dispensed into each well. Plates were finally incubated at room temperature (in dark) for 30 min. 100 µl of the stop solution was added into each well to stop the reaction and absorbance was read at 450 nm. The results were expressed as picogram/ml of the TNF-α released. The ELISA was sensitive to 5 picogram/ml of the TNF-α released.

### Assay for NF-κB p50 subunit

Assay for NF-κB/p50 subunit in the nuclear extracts was performed in all the groups by commercially available Transcription Factor Assay kit (Upstate Biotechnology, NY, USA) according to the manufacturer's instructions. This assay combines the principle of the electrophoretic mobility shift assay (EMSA) with the 96-well based enzyme linked immunosorbent assay (ELISA). Briefly, nuclear extracts were prepared from liver tissue using Chemicon's Nuclear Extraction Kit. During the assay, the capture probe, a double stranded biotinylated oligonucleotide containing the consensus sequence for NF-κB was mixed with the nuclear extract in the transcription factor assay buffer provided directly in the streptavidin coated plate and incubated for 2 h at room temperature. Plates were then washed to remove the unbound material. The bound NF-κB transcription factor subunit p50 was detected with specific primary antibody, rabbit anti-NFκB p50. HRP conjugated secondary antibody was then used for detection using 3,3′,5,5′-tetramethybenzidine (TMB/E) as the substrate and absorbance was read at 450 nm. Positive and negative controls were also run simultaneously.

### Micronucleus Analysis

Micronuclei analysis was done by the method of Schmid [40]. For this assay, liver tissues were washed with chilled homogenizing buffer (24 mM Sodium-EDTA buffer pH 7.5, containing 75 mM of NaCl). 200 mg of tissue was homogenized in 5.0 ml of this buffer at 500 rpm for 30 seconds. The homogenates were then centrifuged at 3835× *g* for 10 min. The supernatant was removed and fresh homogenizing buffer (0.4 ml) was added to re-suspend the hepatocytes. Small drops of suspension prepared were put at one end of pre-cleaned, grease free microscopic slide. The drops were spread using another glass slide held at an angle of 45°C into a smooth layer. The slides were then air dried. The slides were then stained according to the following sequence: fixed in methanol for 10 min; stained in undiluted May-Grunwald solution for 90 seconds; washed with water; stained with Giemsa (1∶10 diluted) for 15–20 min; rinsed twice in distilled water; blot dried with filter paper; back side of the slides cleaned with methanol; slides then cleared in xylene for 5 min, and finally mounted in DPX (Distyrene Plasticizer Xylene). Minimum of 100 cells were counted per sample for the presence of micronuclei using light microscope at 45x.

### Statistical Analysis

The data were expressed as mean ± S.D. Statistical significance between groups was evaluated using Kruskal-Wallis test followed by the Mann-Witney-U multiple comparison test. The statistical analysis was done using the SPSS software package version 16 for Windows (SPSS Inc., Chicago, IL). In all data analysis, p-values of 0.05 or less (p<0.05) were considered significant.

## Results

### Blood Alcohol Levels

In each of the groups studied, the rats increased their weight at a constant rate; there was no difference in weight gain among the groups. Blood alcohol levels (BAL) 1.5 h and 2.5 h after ethanol administration by gavage in the alcohol group were 290.3±41.2 mg/dL and 275.36±17.6 mg/dL, respectively. BAL in the alcohol treated and catechin supplemented group were 282.24±31.7 and 269.8±21.1 mg/dL after 1.5 and 2.5 h of alcohol administration, respectively.

### Plasma endotoxin levels

All rats had detectable endotoxin levels in their plasma, but chronic alcohol exposure caused significant endotoxemia. The plasma endotoxin level in the control group was 0.12±0.01 EU/ml. The endotoxin levels in the alcohol-fed group were 0.38±0.04 EU/ml, nearly 3 fold higher than control group, and the increase was statistically significant at p<0.001. Plasma endotoxin levels in alcohol-treated and catechin supplemented rats were significantly lower than in alcohol-fed rats (0.274±0.05, p<0.01)

### Clinical Chemistry

No significant change in the levels of liver enzymes (ALT and AST) was observed in any of the groups. However, a significant increase in serum ALP levels (262.48 ± 15.68 IU/L, p<0.01) was observed in alcohol-fed rats as compared to the control group (166.17 ± 21.86 IU/L). The activitiy of serum ALP was decreased significantly in alcohol-fed rats on supplementation with catechin (p<0.01). Catechin *per se* had no effect on liver enzyme levels (Table 1).

**Table 1:** Effect of catechin on hepatic markers in the serum of control and alcohol-administered rats.

Groups	ALT (IU/L)	AST (IU/L)	ALP (IU/L)
**Control**	40.96±4.93	272.27±19.85	166.17±21.86
**CT *per se***	40.07±8.34	266.61±28.36	151.47±33.64
**Alcohol (Alc)**	43.02±7.88	283.47±37.28	262.48±15.68*
**Alcohol + CT**	35.36±6.89	240.17±27.48	180.75±38.6#

Values are expressed as mean ± S.D. of six different observations.

*p<0.01 vs. control and catechin *per se*;

# p<0.01 vs. Alc.

**Figure 1:**
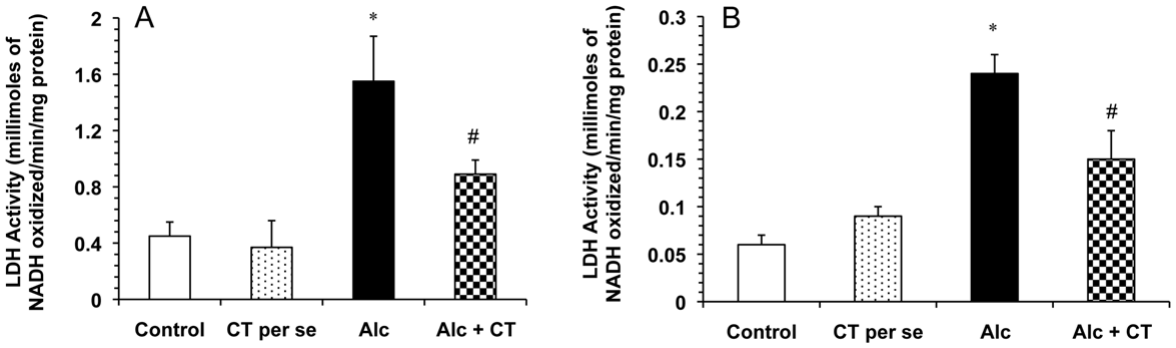
Effect of catechin on LDH activity in alcohol-administered rats. **A**) Serum LDH activity and **B**) hepatic LDH activity. Values are expressed as mean ± S.D. of eight different observations. *p<0.001 vs. control and catechin (CT) *per se*; ^#^p<0.001 vs. Alcohol (Alc).

The activity of LDH in serum and liver homogenates was significantly increased (p<0.001) in the alcohol-fed group as compared with normal control group (1.55±0.32 millimoles/min/mg protein vs. 0.45±0.10 millimoles/min/mg protein in control serum samples; 0.24±0.02 millimoles/min/mg protein vs. 0.06±0.0.01 millimoles/min/mg protein in control homogenates). However, pretreatment with catechin in alcohol-treated rats significantly (p<0.001) reduced the elevations in LDH activity induced by alcohol. Supplementation of catechin in normal rats (*per se*) did not significantly alter elevations (Figures 1A, 1B).

### Hepatic histoarchitecture

**Figure 2:**
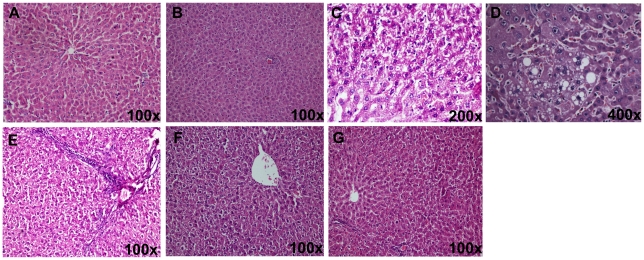
Photomicrographs of hematoxylin-eosin stained rat liver sections after alcohol administration. **A**) Photomicrograph of the normal/control rat liver showing normal liver morphology; **B**) Photomicrograph of rat liver of catechin *per se* group showing normal liver morphology; **C**, **D**) Photomicrograph of the liver from alcohol-administered rat showing vacuolar degeneration, micro- and macrovesicular fatty change; **E**) Photomicrograph of the liver from alcohol-administered rat showing portal triaditis with thin fibrous bridges radiating from the portal tract. Liver cells show vacuolar degeneration and microvesicular fatty change; **F**) Photomicrograph of the liver from alcohol-administered rat supplemented with catechin (Alc + CT) showing mild cytoplasmic vacuolation with no fatty change; **G**) Photomicrograph of the liver from alcohol-administered rat supplemented with catechin (Alc + CT) showing normal liver morphology.

Histological evaluation did not reveal any morphological alterations in the control group (Figure 2A) and catechin *per se* group (Figure 2B). The liver sections of alcohol-administered rats showed vacuolar degeneration, micro and macrofollicular fatty changes, focal collection of lymphocytes and vascular congestion. Portal tract inflammation (portal triaditis) was also observed with thin fibrous bridges radiating from the portal tract (Figures 2C–2E). In contrast, the histological examination of tissue sections from alcohol-fed rats supplemented with catechin showed an improvement of liver morphology except for mild vacuolar degeneration. Necrotic cells and fatty change were nearly absent (Figures 2F, 2G).

### Activation of transcription factor, NF-κB

**Figure 3:**
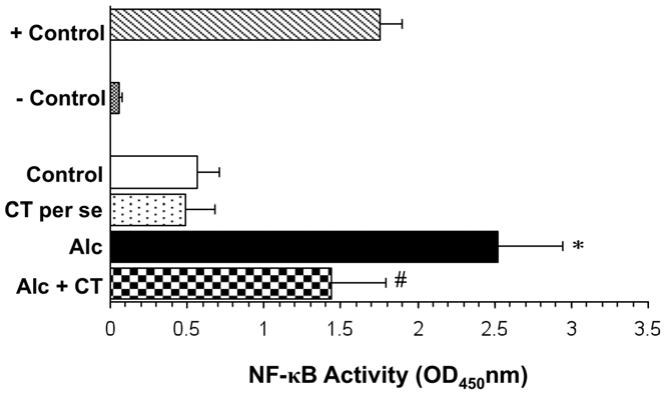
Effect of catechin on alcohol-induced activation of NF-κB in liver. Values are expressed as mean ± S.D. of five different observations. *p<0.001 vs. control and catechin (CT) *per se*; ^#^p<0.01 vs. Alcohol (Alc). Positive control **(+control)** refers to the TNF-α treated Hela whole cell extract; Negative control **(-control)** refers to the biotinylated double stranded non-specific competitor oligonucleotide probe which does not contain the NF-κB consensus sequence.

NF-κB p50 subunit was significantly (p<0.001) elevated in chronically alcohol-fed rats as compared to the control and *per se* groups (Figure 3). Supplementation with catechin significantly attenuated the alcohol-induced activation of NF-κB (p<0.01).

### Hepatic TNF-α levels

**Figure 4:**
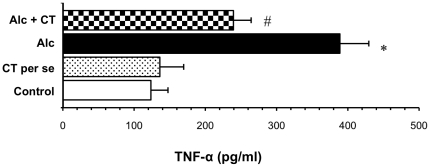
Effect of catechin on hepatic TNF-α levels in alcohol-fed rats. Values are expressed as mean ± S.D. of eight different observations. *p<0.001 vs. control and catechin (CT) *per se*; ^#^p<0.001 vs. Alcohol (Alc).

Chronic alcohol administration resulted in a marked rise in the levels of TNF-α compared to the control group (388.86 ± 40.57 pg/ml vs. 123.58 ± 23.97 pg/ml in control). Supplementation with catechin significantly decreased the levels of TNF-α by 1.62 fold (at p<0.001) (Figure 4).

### Hepatic MDA levels

**Figure 5:**
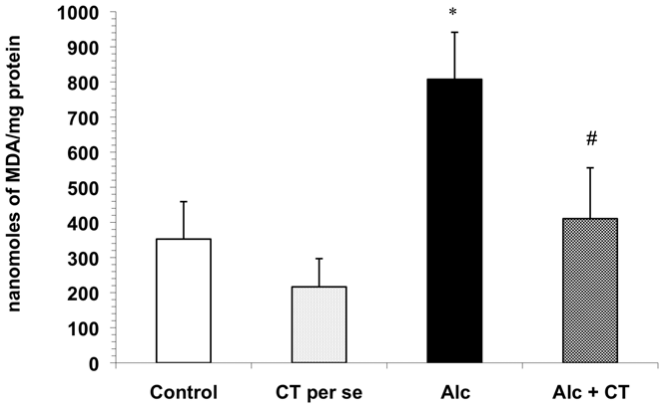
Effect of catechin on liver MDA levels in alcohol-administered rats. Values are expressed as mean ± S.D. of eight different observations. *p<0.001 vs. control and catechin (CT) *per se*; ^#^p<0.001 vs. Alcohol (Alc).

Chronic administration of alcohol led to an increase in hepatic MDA level (807.47±133.95 nanomoles/mg protein) compared to the control group (352.5±106.54 nanomoles/mg protein) indicating an enhancement in the lipid peroxidation potential of the liver (p<0.001) (Figure 5). Although this increase was more than two fold, catechin supplementation to ethanol-treated rats significantly attenuated the alcohol-induced increase (p<0.001) in the liver MDA levels (410.61±144.82 nanomoles/mg protein) as compared to the alcohol group.

### Hepatic antioxidant profile

Alcohol administration caused a significant depletion (p<0.01) of circulatory non-enzymic antioxidant GSH (0.27±0.07 micromoles/mg protein) as compared to control (0.53±0.11 micromoles/mg protein). Catechin co-supplementation to alcoholic rats showed significantly increased levels of reduced glutathione (0.46±0.06 micromoles/mg protein, p<0.001) as compared with alcohol-fed rats (Table 2).

**Table 2:** Effect of catechin on hepatic antioxidant profile in control and alcohol-administered rats.

Parameter	Control	CT *per se*	Alcohol (Alc)	Alcohol + CT
**GSH**	0.53±0.11	0.6±0.13	0.27±0.07*	0.46±0.08#
**SOD**	4.4±0.42	5.03±0.59	1.99±0.17*	3.1±0.28#
**Catalase**	154.52±19.55	165.75±20.93	53.75±17.09*	139.74±18.11#
**GR**	60.35±3.68	65.96±5.25	63.13±4.57	61.14±3.33
**GPx**	49.64±4.68	60.84 ±4.42	54.91±7.25	57.28±6.64

Values are expressed as mean ± S.D. of eight different observations.

*p<0.001 vs. control and catechin *per se*;

# p<0.001 vs. Alc. Units for: **GSH-** micromoles of GSH/mg protein; **SOD-** Units/mg protein; **Catalase-** millimoles of catalase/mg protein; **GR-** micromoles of NADPH oxidized/min/mg protein; **GPx-** micromoles of NADPH oxidized/min/mg protein.

Rats treated chronically with alcohol exhibited a significant decrease in the activities of SOD (2.2 fold, p<0.001) and catalase (2.58 fold, p<0.001) when compared with control rats, whereas GPx and GR activities were not affected. Conversely, catechin supplemented alcoholic rats showed a spectacular restoration of hepatic SOD and catalase activities, which attain near control values when compared to alcohol treated group (p<0.001). Animals in the group supplemented with only catechin (*per se*) did not show any change in the antioxidant enzyme levels (Table 2).

### Nitrite levels

**Figure 6:**
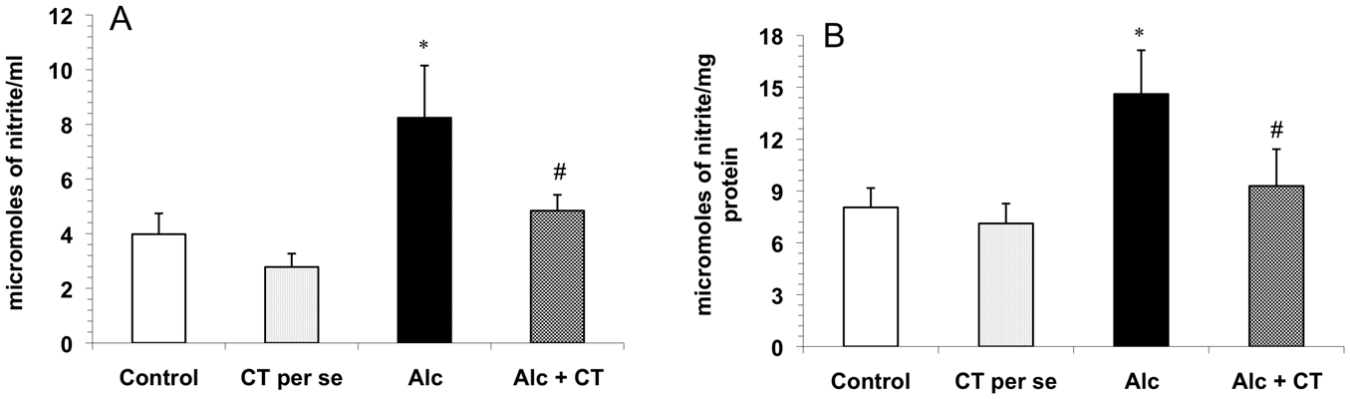
Effect of catechin on A) serum nitrite levels and B) hepatic nitrite levels in alcohol-fed rats. Values are expressed as mean ± S.D. of eight different observations. *p<0.001 vs. control and catechin (CT) *per se*; ^#^p<0.001 vs. Alcohol (Alc).

Nitrite levels in the liver homogenates and serum samples were found to be significantly higher (p<0.001) in chronically alcohol-consuming rats as compared to the control group. Pretreatment with catechin significantly decreased the hepatic and serum nitrite levels (p<0.001) and restored them to near normal levels (Figures 6A and 6B). *Per se* group did not show any significant effect.

### Micronuclei Analysis

**Figure 7:**
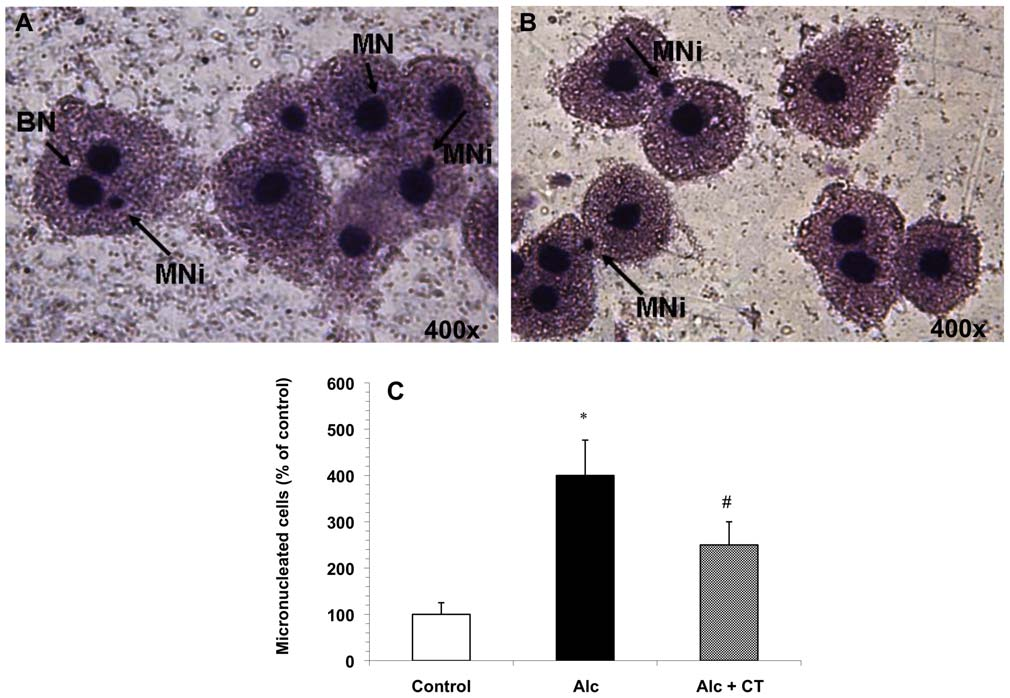
Micronuclei analysis in the hepatocytes of alcohol-fed rats. **A**, **B**) Dividing cells showing normal nuclei and micronuclei in hepatocytes of alcohol-fed rats. **BN**: Binucleated; **MN**: Mononucleus; **MNi**: Micronucleus; **C**) Effect of catechin on the extent of micronuclei formation in hepatocytes of alcohol- administered rats. Values are expressed as percentage of micronucleated cells. *p<0.001 vs. control; ^#^p<0.05 vs. Alcohol (Alc).

A significant increase in the micronucleated cell score was observed after alcohol abuse (Figures 7A, 7B). However, supplementation with catechin to alcohol-fed rats resulted in a significant decrease in micronuclei as compared to the alcohol-administered rats (Figure 7C).

## Discussion

Treatment of alcohol-induced liver disease remains limited to supportive measures [3], [41]. Undoubtedly, the development of effective therapy to prevent or treat ALD will depend on elucidating the suppression/blockage of any of the steps culminating into liver injury. Several lines of evidence suggest that the induction of NF-κB-dependent gene expression in Kupffer cells contributes to alcohol-induced liver injury [42]–[45]. In light of this hypothesis, antioxidants represent a potential group of therapeutic agents for ALD providing beneficial effects against hepatic oxidative stress while inhibiting priming mechanisms for the suppression of NF-κB-dependent target genes for inflammation and cytotoxicity [45]–[48].

In the present study, we observed that catechin, a polyphenolic antioxidant, inhibited the activation of alcohol-induced NF-κB in chronically alcohol-fed rats and prevented necroinflammatory changes (Figures 2 and 3). Although, green tea polyphenols (particularly epigallocatechin gallate) are known to inhibit NF-κB activation in lipopolysaccharide-activated peritoneal macrophages [49], ischemia-reperfusion liver injury [50] and against ethanol-induced gastric mucosal damage [51], this is the first time, that catechin has been shown to inhibit NF-κB thereby preventing ALD. In agreement to our finding, recently, catechin has been shown to reduce hepatic fibrosis by controlling the transcription factor (AP-1) expression that is involved in stellate cell activation [52]. Blocking of NF-κB activation may further downregulate TNF-α, as has been reported earlier [53], [54]. Antibodies to TNF-α attenuated ALD pathology [55], [56] further substantiating that overproduction of TNF-α is an important step in the development of ALD. We also found that catechin significantly decreased the levels of TNF-α in alcohol-administered rats (Figure 4). Decreased levels of TNF-α may thus be attributed to the anti-inflammatory mechanism of catechin. Corroborating with the present data, Yuan and colleagues [17] have reported that green tea polyphenol, epigallocatechin gallate significantly blunted the increase in both CD14 and TNF-α expression in chronically alcohol-fed rat liver.

A significant body of evidence indicates that endotoxemia and endotoxin-mediated hepatocellular damage play a crucial role in the pathogenesis of ALD [6], [57]. Plasma endotoxin levels have been reported to increase in patients with alcoholic hepatitis which correlate with severity of liver disturbance [58]. Kupffer cell activation by endotoxin derived from intestinal bacteria further enhances the generation of TNF-α leading to tissue damage [10]. In the present study, chronic alcohol gavage caused significant endotoxemia in rats which was markedly reduced on supplementation with catechin, thus supporting the correlation amongst endotoxin, Kupffer cells and TNF-α production.

Another factor playing a central role in many pathways of alcohol-induced damage is the excessive generation of free radicals which can lead to oxidative stress. Both acute and chronic alcohol exposure can increase the production of ROS that further enhance peroxidation of lipids, proteins, and DNA, and affect the antioxidant system [59], [60]. In the absence of an appropriate compensatory response from the endogenous antioxidant network, the system may become overwhelmed (redox imbalance), leading to the activation of stress-sensitive signalling pathways, such as NF-κB, and others. Interestingly, cellular sensitivity or resistance to TNF-α is also correlated with decreased or increased levels of SOD respectively [31], [61]. The present study confirms that the levels of TNF-α after alcohol consumption correlated with the increased level of peroxidation and decreased activities of hepatic antioxidants. Here, catechin attenuated the rise of liver MDA levels and increased the levels of hepatic antioxidants after chronic alcohol administration resulting in amelioration of liver injury (Figure 5, Table 2). These effects might have been due to the antioxidative property of the catechin in the supplemented rats. It has been suggested that flavonoids such as catechin could localize near the membrane surface, trapping directly any free radicals generated in the lipid environment of the membranes as well as ROS generated in the aqueous phase [62]. This might block the superoxide radical in the tissues and decrease SOD consumption thus enhancing SOD activity [63]. Moreover, it has been previously shown that green tea flavonoids increase the GSH regulatory elements *in vivo* [64]. Antioxidant compounds and extracts such as green tea and cocoa, which generally reduce ROS production and, consequently, oxidative stress, have also been shown to provide substantial protection against ALD in rats [15], [16], [19].

Chronic alcohol consumption has also been reported to increase nitrite and nitrate levels in animals, as well as in patients with alcoholic liver disease [65], [66]. The increased nitrite and nitrate levels have been reported to be accompanied by increased endotoxin levels in plasma, as has been observed in the present study also. In the liver, nitric oxide (NO) is produced by at least two different isoforms of nitric oxide synthase (NOS), e.g. eNOS and iNOS. eNOS is constitutively expressed and generates relatively small amounts of NO, but plays an important role in vasorelaxation [67]. Overproduction of NO following chronic alcohol consumption is mediated by increased iNOS expression [68] which involves activation of NF-κB as the primary regulatory step [69] NO in conjunction with superoxide radical has been reported to form a potent and versatile oxidant peroxynitrite, which may lead to the stimulation of TNF-α production in Kupffer cells [70]. Enhanced expression of iNOS and hence increased NO levels observed in the chronically alcohol-fed animals might have been associated with TNF-α as it is known for its potent stimulatory activity of iNOS which increases the NO levels [71], [72]. The present study showed that catechin significantly inhibited the hepatic and serum nitrite levels (Figure 6), thus correlating well with the decreased TNF-α levels and NF-κB activity indicating an interplay of these molecules in determining the outcome of a clinical manifestation.

The present study also reports the formation of micronuclei (MNi) in the chronically alcohol-fed rat liver cells. In humans, there is evidence of increased chromosome damage and changes in the number of chromosomes in peripheral blood lymphocytes of heavy drinkers [73]. Significantly, more MNi in the peripheral lymphocytes of alcoholics has been found than in age- and gender-matched controls [74]. MNi originates from acentric chromosome fragments or whole chromosomes that are not included in the main daughter nuclei during nuclear division. Consequently, the frequency of MNi provides a measure of both chromosome breakage and chromosome loss, and can be taken as an indicator of genotoxic response to carcinogenic agents. In the present study, supplementation with catechin decreased the percentage of micronucleated cells in the chronically alcohol-fed rat liver, thereby indicating reduced toxicity (Figure 7).

The results of the functional tests together with histopathological observation also suggest that alcohol leads to serious changes in the hepatic histoarchitecture. In the present study, alcohol administration produced a spectrum of histological abnormalities in the liver, as has been described earlier [75]. These changes were accompanied by substantial elevated levels of ALP and LDH in the serum, indicating the increased permeability and damage and/or necrosis of hepatocytes (Table 1, Figure 1). However, no significant change in the serum AST and ALT levels was observed in the present study. In support of our findings, it has been reported that serum aminotransferase levels may or may not be elevated during chronic alcohol consumption, and the absolute degree of enzyme elevation does not provide much insight into the severity of underlying hepatic inflammation [76]. Catechin supplementation significantly attenuated liver injury as evidenced by a marked reduction in the serum enzyme levels of ALP and LDH and also the restoration of the hepatic histoarchitecture, which is consistent with previous studies with green tea extract [15], [77]. Hence, it is suggested that leakage of enzymes from the hepatocellular membrane is decreased by the membrane stabilizing action of catechin.

In summary, we demonstrate in the present study that catechin ameliorates alcohol-induced liver injury. Recently, we have shown down-regulation of NF-κB signalling by polyphenolic compounds, catechin and quercetin against endotoxin-induced liver injury in a rat model [78]. Since, alcohol induced liver injury is, partially, endotoxin mediated, therefore, the same mechanism seems to be operative here also thereby confirming our earlier findings [78]. The mechanism may involve suppression of alcohol-induced endotoxemia thereby downregulating endotoxin-induced activation of NF-κB and further going downstream the signalling cascade including TNF-α, NO and ROS and by enhancing the antioxidant profile of the chronically alcohol-fed host. Our findings suggest that polyphenols such as catechin that allow transient NF-κB inhibition without incurring prohibitive toxicity or loss of innate immunity, may be of importance in making strategies for management of the clinical manifestations involving oxidative damage. It may also be inferred that catechin, if not alone, may at-least be used in conjunction with the conventional drugs used for the treatment of ALD. This may lower the effective dose of drugs used for the treatment, thereby reducing the associated side-effects besides conferring health benefits to the individuals.

## References

[1] Gramenzi A, Caputo F, Biselli M, Kuria F, Loggi E (2006). Review article: alcoholic liver disease-pathophysiological aspects and risk factors.. Aliment Pharmacol Ther.

[2] Tilg H, Day CP (2007). Management strategies in alcoholic liver disease.. Nature Clin Prac: Gastroenterol Hepatol.

[3] Barve A, Khan R, Marsano L, Ravindra KV, McClain C (2008). Treatment of alcoholic liver disease.. Ann Hepatol.

[4] Lieber CS (2004). Alcoholic fatty liver: its pathogenesis and mechanism of progression to inflammation and fibrosis.. Alcohol.

[5] Lucey MR, Mathurin P, Morgan TR (2009). Alcoholic hepatitis.. N Engl J Med.

[6] Rao RK, Seth A, Sheth P (2004). Recent advances in alcoholic liver disease I. Role of intestinal permeability and endotoxemia in alcoholic liver disease.. Am J Physiol Gastrointest Liver Physiol.

[7] Baeuerle PA, Baltimore D (1996). NF-κB: ten years after.. Cell.

[8] Barnes PJ, Karin M (1997). Nuclear factor κB-a pivotal factor in chronic inflammatory diseases.. N Engl J Med.

[9] Nanji AA, Jokelainen K, Rahemtulla A, Miao L, Fogt F (1999). Activation of nuclear factor kappa B and cytokine imbalance in experimental alcoholic liver disease in rat.. Hepatology.

[10] Wheeler MD (2003). Endotoxin and Kupffer cell activation in alcoholic liver disease.. Alcohol Res Health.

[11] Schaffert CS, Duryee MJ, Hunter CD, Hamilton BC, DeVeneyet AL (2009). Alcohol metabolites and lipopolysaccharide: Roles in the development and/or progression of alcoholic liver disease.. World J Gastroenterol.

[12] Ozaras R, Tahan V, Aydin S, Uzun H, Kaya S (2003). N-acetylcysteine attenuates alcohol-induced oxidative stress in the rat.. World J Gastroenterol.

[13] Kasdallah-Grissa A, Mornagui B, Aouani E, Hammami M, May ME (2007). Resveratrol, a red wine polyphenol, attenuates ethanol-induced oxidative stress in rat liver.. Life Sci.

[14] Chen Xi (2010). Protective effects of quercetin on liver injury induced by ethanol.. Pharmacogn Mag.

[15] Arteel GE, Uesugi T, Bevan LN, Gäbele E, Wheeler MD (2002). Green tea extract protects against early alcohol-induced liver injury in rats.. Biol Chem.

[16] McKim SE, Konno A, Gabele E, Uesugi T, Froh M (2002). Cocoa extract protects against early alcohol-induced liver injury in the rat.. Arch Biochem Biophys.

[17] Yuan G, Gong Z, Zhou X, Zhang P, Sun X (2006). Epigallocatechin-3-gallate ameliorates alcohol-induced liver injury in rats.. Int J Mol Sci.

[18] Zhang XG, Xu P, Liu Q, Yu CH, Zhang Y (2006). Effect of tea polyphenols on cytokine gene expression in rats with alcoholic liver disease.. Hepatobiliary Pancreat Dis Int.

[19] Kaviarasan S, Sundarapandiyan R, Anuradha CV (2008). Epigallocatechin gallate, a green tea phytochemical, attenuates alcohol-induced hepatic protein and lipid damage.. Toxicol Mech Methods.

[20] Rice-Evans C, Miller NJ, Paganga G (1996). Structure-antioxidant activity relationships of flavonoids and phenolic acids.. Free Radic Biol Med.

[21] Bravo L (1998). Polyphenols: chemistry, dietary sources, metabolism and nutritional significance.. Nutr Rev.

[22] Jovanovic SV, Steenken S, Simic MG, Hara Y, Rice-Evans C, Packer L (1998). Antioxidant properties of flavonoids: reduction potentials and electron transfer reactions of flavonoid radicals.. Flavonoids in health and disease.

[23] Gonzalez-Gallego J, Sanchez-Campos S, Tunon MJ (2007). Anti-inflammatory properties of dietary flavonoids.. Nutr Hosp.

[24] Wang H, Provan GJ, Helliwell K (2000). Tea flavonoids: Their functions, utilization and analysis.. Trends Food Sci Technol.

[25] Higdon JV, Frei B (2003). Tea catechins and polyphenols: health effects, metabolism, and antioxidant functions.. Crit Rev Food Sci Nutr.

[26] Yuan GJ, Gong ZJ, Sun XM, Zheng SH, Li X (2006). Tea polyphenols inhibit expressions of iNOS and TNF-α and prevent lipopolysaccharide-induced liver injury in rats.. Hepatobiliary Pancreat Dis Int.

[27] Zhen MC, Wang Q, Huang XH, Cao LQ, Chen XL (2007). Green tea polyphenol epigallocatechin-3-gallate inhibits oxidative damage and preventive effects on carbon-tetrachloride-induced hepatic fibrosis.. J Nutr Biochem.

[28] Colman JC, Morgan MY, Scheuer PJ, Sherlock S (1980). Treatment of alcohol-related liver disease with (+)-cyanidanol-3: a randomised double-blind trial.. Gut.

[29] World MJ, Ryle PR, Aps EJ, Shaw GK, Thomson AD (1987). Palmitoyl-catechin for alcoholic liver disease: Results of a three-month clinical trial. Alcohol Alcohol.

[30] Wills ED (1966). Mechanisms of lipid peroxide formation in animal tissues.. Biochem J.

[31] Chanana V, Majumdar S, Rishi P (2007). Involvement of caspase-3, lipid peroxidation and TNF-alpha in causing apoptosis of macrophages by coordinately expressed Salmonella phenotype under stress conditions.. Mol Immunol.

[32] Lowry OH, Rosenbrough NJ, Farr AL, Randall RJ (1951). Protein measurement with Folin's phenol reagent.. J Biol Chem.

[33] Jollow D, Mitchell L, Zampaglione N, Gillette JR (1974). Bromobenzene induced liver necrosis: protective role of glutathione and evidence for 3, 4-bromobenzenoxide as the hepatotoxic intermediate.. Pharmacol.

[34] Kono Y (1978). Generation of superoxide radical during autooxidation of hydroxylamine and an assay for superoxide dismutase.. Arch Biochem Biophys.

[35] Luck H, Bergmeyer HU (1971). Catalase.. Methods of Enzymatic Analysis.

[36] Paglia DE, Valentine WN (1976). Studies on the qualitative and quantitative characterization of erythrocyteglutathione peroxidase.. J Lab Clin Med.

[37] Carlberg I, Mannervik B (1975). Purification and characterization of the flavoenzyme glutathione reductase from rat liver.. J Biol Chem.

[38] Bergmeyer HU, Bernt E, Hess B, Bergmeyer HU (1971). Lactate dehydrogense.. Methods of Enzymatic Analysis.

[39] Green LC, Wagner DA, Glogowski J, Skipper PL, Wishnok JS (1982). Analysis of nitrate, nitrite and 15N nitrate in biological fluids.. Anal Biochem.

[40] Schmid S (1975). An evaluation of the micronuclei test using triethylenemelamine, trimethylphosphate and niridazole.. Mutat Res.

[41] Mullen KD, Dasrathy S (1998). Potential new therapies for alcoholic liver disease.. Clin Liver Dis.

[42] Szabo G (2000). New insights into the molecular mechanisms of alcoholic hepatitis: a potential role for NF-κB activation?. J Lab Clin Med.

[43] Tsukamoto H, Lu SC (2001). Current concepts in the pathogenesis of alcoholic liver injury.. FASEB J.

[44] Uesegi T, Froh M, Arteel GE, Bradford BU, Gabele E (2001). Delivery of IκB superrepressor gene with adenovirus reduces early alcohol-induced liver injury in rats.. Hepatology.

[45] Beier JI, McClain CJ (2009). Mechanisms and cell signalling in alcoholic liver disease.. Biol Chem.

[46] Hill DB, Devalaraja R, Joshi-Barve S, McClain CJ (1999). Antioxidants attenuate nuclear factor-kappa B activation and tumor necrosis factor-alpha production in a alcoholic hepatitis patient monocytes and rat Kupffer cells, in vitro.. Clin Biochem.

[47] Yamamoto Y, Gaynor RB (2001). Therapeutic potential of inhibition of NF-κB pathway in the treatment of inflammation and cancer.. J Clin Invest.

[48] Nanji AA, Jokelainen K, Tipoe GL, Rahemtulla A, Thomas P (2003). Curcumin prevents alcohol-induced liver disease in rats by inhibiting the expression of NF-κB-dependent genes.. Am J Physiol Gastrointest Liver Physiol.

[49] Lin YL, Lin JK (1997). (-)-Epigallocatechin-3-gallate blocks the induction of nitric oxide synthase by down-regulating lipopolysaccharide-induced activity of transcription factor nuclear factor-kappaB.. Mol Pharmacol.

[50] Zhong Z, Froh M, Connor HD, Li X, Conzelmann LO (2002). Prevention of hepatic ischemia-reperfusion injury by green tea extract.. Am J Physiol Gastrointest Liver Physiol.

[51] Lee JS, Oh TY, Kim YK, Baik JH, So S (2005). Protective effects of green tea polyphenol extracts against ethanol-induced gastric mucosal damages in rats: Stress-responsive transcription factors and MAP kinases as potential targets.. Mutat Res.

[52] Kobayashi H, Tanaka Y, Asagiri K, Asakawa T, Tanikawa K (2010). The antioxidant effect of green tea catechin ameliorates experimental liver injury.. Phytomedicine.

[53] Su GL (2002). Lipopolysaccharides in liver injury: molecular mechanisms of Kupffer cell activation.. Am J Physiol.

[54] Hines IN, Wheeler MD (2004). Recent advances in alcoholic liver disease III. Role of the innate immune response in alcoholic hepatitis.. Am J Physiol Gastrointest Liver Physiol.

[55] Imuro Y, Gallucci RM, Luster ML, Kono H, Thurman RG (1997). Antibodies to tumor necrosis factor alpha attenuate hepatic necrosis and inflammation caused by chronic exposure to ethanol in the rat.. Hepatology.

[56] Yin M, Wheeler MD, Kono H, Bradford BU, Gallucci RM (1999). Essential role of tumor necrosis factor alpha in alcohol-induced liver injury.. Gastroenterology.

[57] Thurman RG (1998). Mechanisms of hepatic toxicity II. Alcoholic liver injury involves activation of Kupffer cells by endotoxin.. Am J Physiol Gastrointest Liver Physiol.

[58] Fujimoto M, Uemura M, Nakatani Y, Tsujita S, Hoppo K (2000). Plasma endotoxin and serum cytokine levels in patients with alcoholic hepatitis: relation to severity of liver disturbance.. Clin Exp Res.

[59] Nordmann R (1994). Alcohol and antioxidant systems.. Alcohol Alcohol.

[60] Wu D, Cederbaum AI (2009). Oxidative stress and alcoholic liver disease.. Semin Liver Dis.

[61] Hirose K, Longo DL, Oppenheim JJ, Matsushima K (1993). Over expression of mitochondrial manganese superoxide dismutase promotes the survival of tumor cells exposed to interleukin-1, tumor necrosis factor, selected anti cancer drugs, and ionizing radiation.. FASEB J.

[62] Salah N, Miller NJ, Paganga G, Tijburg L, Bolwell GP (1995). Polyphenolic flavanols as scavengers of aqueous phase radicals and as chain-breaking antioxidants.. Arch Biochem Biophys.

[63] Kim SJ, Han D, Moon KD, Rhee JS (1995). Measurement of superoxide dismutase-like activity of natural antioxidants.. Biosci Biotechnol Biochem.

[64] Frei B, Higdon JV (2003). Antioxidant activity of tea polyphenols *in vivo*: evidence from animal studies.. J Nutr.

[65] Guarner C, Soriano G, Thomas A, Bulbena O, Novella MT (1993). Increased serum nitrite and nitrate levels in patients with cirrhosis: relationship to endotoxemia.. Hepatology.

[66] Wang JF, Greenberg SS, Spitzer JJ (1995). Chronic alcohol administration stimulates nitric oxide formation in the rat liver with or without pretreatment by lipopolysaccharide.. Alcohol Clin Exp Res.

[67] Chen T, Zamora R, Zuckerbraun B, Billiar TR (2003). Role of nitric oxide in liver injury.. Curr Mol Med.

[68] McKim SE, Gabele E, Isayama F, Lambert JC, Tucker LM (2003). Inducible nitric oxide synthase is required in alcohol-induced liver injury: studies with knockout mice.. Gastroenterology.

[69] Bogdan C (2001). Nitric oxide and the immune response.. Nature Immunol.

[70] Matata BM, Galinanes M (2002). Peroxynitrite is an essential component of cytokines production mechanism in human monocytes through modulation of nuclear factor-kappaB DNA binding activity.. J Biol Chem.

[71] Song K, Chen Y, Göke R, Wilmen A, Seidel C (2000). Tumor necrosis factor related apoptosis-inducing ligand (TRAIL) is an inhibitor of autoimmune inflammation and cell cycle progression.. J Exp Med.

[72] Chanana V, Majumdar S, Rishi P (2006). Tumour necrosis factor-α mediated apoptosis in murine macrophages by Salmonella enterica serovar Typhi under oxidative stress.. FEMS Immunol Med Microbiol.

[73] Hutter E, Matthies U, Nikolova T, Ehrenreich H (1999). A follow-up study on chromosomal aberrations in lymphocytes of alcoholics during early, medium and long-term abstinence.. Alcohol Clin Exp Res.

[74] Castelli E, Hrelia P, Maffei F, Fimognari C, Foschi FG (1999). Indicators of genetic damage in alcoholics: reversibility after alcohol abstinence.. Hepatogastroenterol.

[75] MacSween RN, Burt AD (1986). Histologic spectrum of alcoholic liver disease.. Semin Liver Dis.

[76] Diehl AM (2002). Liver disease in alcohol abusers: clinical perspective.. Alcohol.

[77] Baltaziak M, Skrzydlewska E, Sulik A, Famulski W, Koda M (2004). Green tea as an antioxidant which protects against alcohol induced injury in rats-a histopathological examination.. Folia Morphol.

[78] Bharrhan S, Chopra K, Arora SK, Toor JS, Rishi P (2011). Down-regulation of NF-{kappa}B signalling by polyphenolic compounds prevents endotoxin-induced liver injury in a rat model.. Innate Immun 14. [Epub ahead of print].

